# Comparative efficacy of oral and cloacal administration of *Lactobacillus* probiotics and postbiotics against *Campylobacter jejuni* colonization in broiler chickens

**DOI:** 10.1016/j.psj.2026.106576

**Published:** 2026-02-02

**Authors:** Shreeya Sharma, Hosni Hassan, Khaled Abdelaziz

**Affiliations:** a Department of Animal and Veterinary Science, Clemson University, Clemson, SC 29634, USA; b Prestage Department of Poultry Science, North Carolina State University, NC 27695, USA; c Clemson University School of Health Research (CUSHR), Clemson, SC 29634, USA

**Keywords:** Campylobacter, Probiotics, Postbiotics, Chicken, Lactobacillus

## Abstract

*Campylobacter jejuni* remains a major cause of foodborne illness worldwide, with poultry serving as the primary reservoir. In the absence of commercial vaccines or effective feed additives, probiotics and their byproducts (postbiotics) represent a promising and sustainable approach to reducing *Campylobacter* colonization in poultry. This study compared the efficacy of oral and cloacal administration of probiotic lactobacilli and their postbiotics in reducing *Campylobacter* colonization and modulating the cecal microbiome in broiler chickens. Day-old chicks were assigned to seven treatment groups that received either probiotics (live cells of four poultry-derived *Lactobacillus* strains: *L. reuteri* P43, *L. acidophilus* P42, *L. animalis* P38, and *L. crispatus* C25) or postbiotics (*Lactobacillus* supernatants) or their combination (whole cultures) orally or intracloacally, with a non-treated group serving as a control. Chickens were challenged with *C. jejuni* strain 81-176 at the second week of age, and cecal contents were collected at the fifth week for *Campylobacter* enumeration and microbiome profiling. The results revealed that both oral and cloacal administration of *Lactobacillus* cells significantly reduced *Campylobacter* cecal loads by 0.34 and 0.78 log₁₀, respectively, compared to the control. Significant differences in microbial richness and evenness were observed among treatment groups, with groups administered orally with probiotics, postbiotics, or their combination consistently showing higher alpha diversity indices than controls. NMDS ordination confirmed distinct community clustering among the treatment groups. Differential abundance analysis (MaAsLin2) further revealed that *Ruminococcus* was significantly enriched in the group receiving intracloacal postbiotic treatment, whereas the genus *unclassified Firmicutes* was more abundant in the group that received the combined probiotic–postbiotic treatment orally. Opportunistic genera, such as *Escherichia-Shigella* and *Faecalicoccus,* were significantly higher in the control group compared to all treated groups. Overall, while probiotics and postbiotics, whether given alone or together, modulated the gut microbial composition in *Campylobacter*-infected broilers, the administration of probiotic cells offered additional benefits by reducing *Campylobacter* colonization.

## Introduction

*Campylobacter jejuni* is one of the leading causes of bacterial foodborne gastroenteritis worldwide (Centre for Disease Control and Prevention, 2025a; Kaakoush et al., 2015). Poultry serves as a major reservoir and a primary source for human *Campylobacter* infections through the handling and consumption of contaminated poultry products (Al Hakeem et al., 2022; Taha-Abdelaziz et al., 2023). In the US, *Campylobacter* accounts for an estimated 1.5 million cases annually (Centre for Disease Control and Prevention, 2025b). Surveillance data show that *Campylobacter* was detected in approximately 30 to 50% of retail chicken samples (Kostoglou et al., 2023; NARMS, 2020), reflecting widespread colonization of commercial broiler flocks and associated public health risk. Reducing *Campylobacter* levels in live birds before slaughter is therefore critical for improving food safety (Arsi et al., 2015b; Taha-Abdelaziz et al., 2023).

While several feed-based interventions have been investigated to enhance colonization resistance to *Campylobacter* in broiler chickens, probiotics have emerged as a key strategy due to their diverse benefits to the host (Al Hakeem et al., 2022; Guyard-Nicodème et al., 2016; Kaakoush et al., 2015; Taha-Abdelaziz et al., 2023). Probiotics are “live microorganisms that confer health benefits to the host when administered in adequate amounts” (FAO/WHO, 2002). In poultry, probiotics, particularly lactic acid bacteria (LAB), have been shown to improve gut health by modulating the immune system, strengthening epithelial barrier integrity, and supporting a diverse, metabolically active microbiome through multiple actions, such as competitive exclusion of pathogenic bacteria and secretion of antagonistic compounds such as short-chain fatty acids (SCFA), organic acids and bacteriocins (FAO/WHO, 2002; Hardy et al., 2013; Kulkarni et al., 2019; Patterson & Burkholder, 2003; Sanders et al., 2010). However, while probiotics have been extensively explored as modulators of the gut microbiota, their efficacy against *Campylobacter* remains variable (Cui et al., 2024; Deng et al., 2020; Saint-Cyr et al., 2016; Shrestha et al., 2017; Śmiałek et al., 2021; Taha-Abdelaziz et al., 2019). Aside from differences in probiotic strain, chicken breed, and dosing regimen, another possible explanation for the observed variability is that certain probiotic strains may fail to provide benefits because they cannot survive the harsh conditions during passage through the digestive tract. Orally administered probiotics must survive the low pH and digestive enzymes of the crop and proventriculus before reaching the intestine, which can drastically reduce viable counts and their effectiveness (Krysiak et al., 2021).

Cloacal inoculation of probiotics has been proposed as a strategy to bypass the upper digestive tract entirely (Arsi et al., 2015a) and to directly deliver probiotic cultures to the lower gut, including the ceca, the primary site of *Campylobacter* colonization. Previous work using ten *Bacillus*-based probiotic isolates showed minimal impact on *Campylobacter* colonization when given orally; only one isolate showed a 1-log_10_ reduction in cecal *Campylobacter* counts, whereas six isolates achieved 1–3 log_10_ reductions when delivered cloacally (Arsi et al., 2015a).

In addition to live probiotic bacteria, there is growing interest in postbiotics as a novel intervention. Postbiotics are defined as “preparation of inanimate microorganisms and/or their components that confer a health benefit on the host.” (Salminen et al., 2021). Postbiotic preparations contain bioactive byproducts: organic acids, bacteriocins, peptides, short-chain fatty acids (SCFAs) and enzymes that can modulate the gut environment and inhibit pathogens (Aguilar-Toalá et al., 2018; Scott et al., 2022; Thorakkattu et al., 2022). As postbiotics contain inanimate cells and/or their byproducts rather than live organisms, they may offer advantages in terms of handling, storage and stability compared to live probiotics, while maintaining functional efficacy (Mosca et al., 2022; Zdybel et al., 2025).

*Campylobacter* is highly sensitive to the acidic and antagonistic environment created by lactobacilli byproducts (Neal-McKinney et al., 2012; Taha-Abdelaziz et al., 2019). Several studies have demonstrated that postbiotics from *Lactobacillus* spp.**,** particularly *L. salivarius, L. reuteri*, and *L. acidophilus*, exert strong inhibitory effects against *Campylobacter* with reductions exceeding 5–6 log₁₀ cfu/mL *in vitro* (Kizerwetter-Swida & Binek, 2009; Mortada et al., 2020; Neal-McKinney et al., 2012; Saint-Cyr et al., 2017; Taha-Abdelaziz et al., 2019).

In light of these findings, this study was conducted to compare the efficacy of poultry-derived multi-strain lactobacilli (*L. reuteri, L. acidophilus, L. animalis*, and *L. crispatus*), their postbiotics and the combination of probiotics and postbiotics, administered either orally (via gavage) or intracloacally (via cloaca) in reducing *C. jejuni* colonization and modulating the cecal microbiota composition in broiler chickens.

## Materials and methods

### Ethics statement

All procedures were conducted in compliance with relevant guidelines and regulations, as approved by the Clemson University Institutional Animal Care and Use Committee (IACUC) under protocol number AUP2022-0411, and in accordance with the ARRIVE 2.0 guidelines.

### Preparation of the lactobacilli and *C. jejuni* cultures

Poultry-derived *Lactobacillus* strains (*L. reuteri* P43, *L. acidophilus* P42, *L. animalis* P38, and *L. crispatus* C25), isolated from the ceca of 4-week-old layer birds and characterized by Dr. Hosni Hassan’s laboratory at North Carolina State University (Hassan et al., 2020, 2024; Rezvani et al., 2016b, 2016a), were cultured under anaerobic conditions. Briefly, a loopful of each frozen strain was inoculated into 10 mL of de Man, Rogosa, and Sharpe (MRS) broth (BD Difco™ Lactobacilli MRS Broth, BD Diagnostic Systems, NJ) and incubated at 37°C for 16 to 18 h in a gas jar containing BD GasPak™ anaerobic packs. Subsequently, 500 µL (1 %) of each overnight culture was transferred into 50 mL of fresh MRS broth and incubated anaerobically at 37°C for 24 h. The cultures were centrifuged at 3,500 × *g* for 10 min at 4°C, and both the cell pellets and supernatants were collected.

Three types of *Lactobacillus*-based preparations were used: *Lactobacillus* cells (probiotics), *Lactobacillus* cell-free supernatant (postbiotic), and whole culture (combination of probiotic cells and their postbiotic supernatant). The supernatants were filtered through 0.45 µm syringe filters and pooled in equal volumes to form the *Lactobacillus* postbiotic preparation. To verify that no viable bacteria remained in the postbiotic supernatant, aliquots were streaked onto MRS agar and incubated; no colony growth was observed. For the probiotic cells, the pellets were washed twice with sterile 1 × PBS, resuspended, and adjusted to 10⁶ cfu/mL in PBS for each strain before pooling in equal proportions. The *Lactobacillus* concentration of 10⁶ cfu/mL was determined in our earlier studies (Sharma et al., 2024; Sharma, et al., 2025). For the whole-culture preparation, the uncentrifuged MRS cultures were adjusted to 10⁶ cfu/mL using fresh sterile MRS broth.

*C. jejuni* strain 81–176 was cultured on Brain Heart Infusion (BHI) agar supplemented with Preston *Campylobacter* selective supplement (Oxoid, UK) under microaerophilic conditions (5 % O₂, 10 % CO₂, 85 % N₂) using BD GasPak™ CampyGen™ gas packs at 37°C for 40 h. Fresh colonies were collected from the agar plates and inoculated into BHI broth for overnight incubation under the same microaerophilic conditions to obtain an actively growing culture. The bacterial cells were then harvested by centrifugation at 3,500 × *g* for 10 min at 4°C, washed twice with sterile 1 × PBS, and resuspended to achieve a final concentration of approximately 1 × 10⁷ cfu/mL in 1 × PBS for oral challenge.

### Experimental design

One-day-old Ross 308 broiler chicks (n = 70) were obtained from Fieldale Farms, GA and reared at the Godley Snell Research Center, Clemson University, SC. Chicks were randomly allocated into seven experimental groups (n = 10/group): postbiotic-PO (per os; group A), postbiotic-IC (intracloacally; group B), whole culture-PO (group C), whole culture-IC (group D), cell-PO (group E), cell-IC (group F), and a non-treated control group (G), based on the treatment and route of administration, as outlined in Table 1. Environmental conditions, including temperature and humidity, were monitored daily. The individual chick was considered the experimental unit for statistical analyses. Treatment group was considered as the main fixed effect. All treatment groups were housed in the same room, and the control group was kept in an adjacent, separate room under identical management conditions. Oral and cloacal administration of probiotics and postbiotics preparations was carried out on the first day of hatch and then on a weekly basis thereafter up to five weeks of age. Oral administration was performed via gavage directly into the crop using sterile insulin syringes, while cloacal administration was performed by gentle insertion of the syringe tip into the cloaca. Each chick in the treatment groups received 1 mL of the prepared suspension, administered either orally or via the cloaca according to the assigned treatment. On day 15 of age, each bird was orally challenged with *C. jejuni* at 10⁷ cfu/mL.

**Table 1:** Experimental design of the study.

Group	Treatment			*Campylobacter* challenge	Necropsy (n=10)
	*Lactobacillus* preparation	Route	Frequency		
Postbiotic-PO (A)	Cell-free supernatant	PO (oral/per os)	Weekly (from wk 1-5)	Day 15 of age (10^7^ cfu/mL)	Day 35 of age (cecal contents)
Postbiotic-IC (B)	Cell-free supernatant	IC (intracloacal)			
Whole-PO (C)	Whole culture (probiotic cells + postbiotic)	PO			
Whole-IC (D)	Whole culture (probiotic cells + postbiotic)	IC			
Cells-PO (E)	Probiotic cells	PO			
Cells-IC (F)	Probiotic cells	IC			
Control (G)	-	-	-		

### Sample collection

Chickens were humanely euthanized at day 35 of age using carbon dioxide (CO₂), in accordance with the AVMA Guidelines. Death was confirmed by the application of bilateral pneumothorax as a secondary physical method, in accordance with Clemson University Institutional Animal Care and Use Committee (IACUC) guidelines. 0.5-1 g of cecal contents was collected from ten birds per group by squeezing both ceca into sterile bacterial culture tubes containing 4.5 mL of 1 × PBS for *Campylobacter* enumeration and from eight birds per group into sterile 1.5 mL tubes for microbiome analysis. All tubes were immediately placed on wet (crushed) ice following collection, and samples for microbiome analysis were subsequently stored at −80°C.

### *Campylobacter* colony counts

Cecal contents collected from individual birds were serially diluted tenfold (10⁻¹ to 10⁻⁷), and each dilution was spread on Brain Heart Infusion (BHI) Agar (BD Diagnostics, NJ) supplemented with Preston selective supplement (Oxoid, UK). Subsequently, plates were incubated in microaerophilic conditions (approx. 5 % O₂) at 37°C for 48 h using CampyGen gas packs (Oxoid, UK). *Campylobacter* colonies were counted and reported as log₁₀ cfu per g of cecal content, as previously described (Taha-Abdelaziz et al., 2018a).

### DNA extraction

Total DNA was extracted from around 300 to 400 mg of cecal content using the QIAmp® PowerFecal® Pro DNA Kit (Qiagen, MD). DNA mass and purity were measured using the NanoDrop One Spectrophotometer (Thermofisher Scientific, MA), ensuring that the 260/280 and 260/230 ratios of the genomic DNA fell within the range of 1.8-2.

### Amplicon library preparation and sequencing

Two microliters of genomic DNA were added to a 96-well PCR plate (Bio-Rad, CA) and submitted to the Clemson University Genomics and Bioinformatics Facility, SC, for library preparation. The procedures outlined by Kozich et al. (2013) were followed, with slight modifications, to amplify the V3-V4 region of the 16S rRNA gene. The forward primer is 5′ CCTACGGGAGGCAGCAG 3′ and the reverse primer is 5′ GGACTACHVGGGTWTCTAAT 3′.

Amplifications were carried out in 20-µL PCR reactions, which included 1 µL of DNA, 5 µL of a unique dual index primer set, 2 µL of 10x Accuprime PCR Buffer II (Invitrogen, CA), 0.15 µL of Accuprime HiFi polymerase, and 11.85 µL of pure water per reaction. The PCR reactions were performed using a BioRad CFX Connect (BioRad, CA) with the following conditions: an initial denaturation stage of 2 min at 95°C, followed by 30 cycles of denaturation at 95°C for 20 seconds, annealing at 55°C for 15 seconds, and extension at 72°C for 5 min. The reactions concluded with a final extension at 72°C for 10 min. Amplification was confirmed using a 2 % agarose E-gel (Invitrogen, CA) to ensure the presence of amplicon libraries. Libraries were then normalized using a SequalPrep Normalization Plate Kit (ThermoFisher, MA) prior to pooling. The pooled DNA samples were quantified using quantitative PCR (qPCR) with the NEBNext Library Quant Kit for Illumina (New England Biolabs, MA). Sequencing was performed using the Illumina NextSeq 2000 platform, which sequenced the multiplexed pooled libraries in paired-end mode (2×150 cycles) to an average depth of 455812. Post-sequencing, samples were demultiplexed using bcl2fastq v4.2.7. An extraction blank (no biological material) was processed alongside cecal samples during DNA extraction and library preparation to monitor potential reagent- and workflow-associated contamination as a quality control measure, consistent with recommended practices for poultry microbiota studies (Lyte et al., 2025).

### Bioinformatic analysis

A total of 26,212,889 raw paired-end reads were generated in fastq.gz format using the Illumina NextSeq 2000 platform. Demultiplexing was performed with bcl2fastq2 Conversion Software v2.20 (Illumina, CA). Sequence processing was conducted using mothur v1.48.2, following the MiSeq standard operating procedure described by Kozich et al. (2013), with parameter modifications optimized for chicken cecal samples. Paired reads were assembled into contigs, screened for quality (parameters: maxambig = 0, minlength = 400, maxlength = 430, maxhomop = 8), and aligned to the SILVA reference database v138 trimmed to the V3–V4 region (positions 6388-25318). Sequences were dereplicated, filtered to informative columns, and pre-clustered (diffs = 4) to reduce sequencing noise. Chimeras were identified and removed using the VSEARCH algorithm (dereplicate = T).

Taxonomic classification was performed using the RDP classifier (trainset v16) with an 80 % bootstrap cutoff. Sequences classified as Chloroplast, Mitochondria, Archaea, Eukaryotes, or unknown were excluded. The resulting dataset contained 459,228 unique Amplicon Sequence Variants (ASVs), from which low-abundance ASVs (< 5 total reads across the dataset) were filtered out, yielding 16,489 ASVs for downstream analyses.

All downstream analyses and visualizations were conducted in R v4.4.0, using the phyloseq (v1.48.0), vegan (v2.6-6), dplyr (v1.1.4), ggplot2 (v3.5.1), rstatix (v0.7.2), ggsignif (v0.6.4), MaAsLin2 (v1.14.1) and tidyverse (v2.0.0) packages.

### Statistical analysis

Alpha diversity (Shannon, Simpson, and observed ASVs) was calculated using the summary.single() command in mothur and verified in R. The data were tested for normality using the Shapiro–Wilk test. Differences in alpha diversity among groups were analyzed using the Kruskal–Wallis test followed by Dunn’s post hoc test for pairwise comparison. Beta diversity was calculated using Bray–Curtis dissimilarity, with significance assessed by PERMANOVA with 9,999 permutations (using the adonis2 in the vegan package). Differentially abundant genera were identified using MaAsLin2 (Multivariable Association with Linear Models) (Mallick et al., 2021). Benjamini-Hochberg FDR was used as multiple correction testing for MaAsLin2.

The log₁₀-transformed *C. jejuni* counts (cfu/g of cecal content) did not meet assumptions of normality (using the Shapiro–Wilk test), and thus group differences were evaluated using a nonparametric Kruskal–Wallis test. Pairwise comparisons among treatment groups were performed using Dunn’s post hoc test (p < 0.05).

## Results

### Probiotic cells (Groups E and F) significantly lowered cecal *Campylobacter* load

**Fig. 1:**
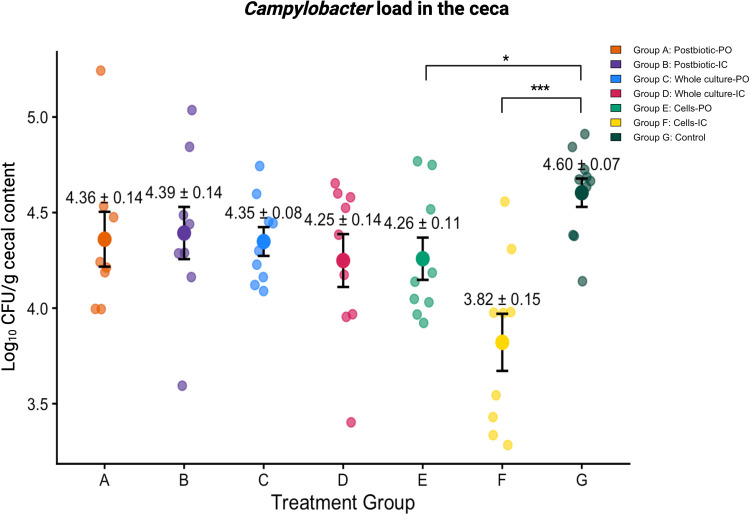
Cecal *Campylobacter* load (cfu/g) across treatment groups. Log₁₀-transformed *Campylobacter* counts (cfu/g cecal content) were quantified in chicks receiving different treatments. Treatment groups included postbiotic administered orally (Group A, postbiotic-PO) or intracloacally (Group B, postbiotic-IC), whole culture probiotic administered orally (Group C, whole culture-PO) or intracloacally (Group D, whole culture-IC), probiotic cells administered orally (Group E, cell-PO) or intracloacally (Group F, cell-IC), and a non-treated control group (Group G). Individual data points represent values from individual birds, while bars indicate group means ± SEM. Statistical significance between groups is indicated by asterisks, where * *p* < 0.05 and *** *p* < 0.001.

Pairwise comparisons of cecal *Campylobacter* load indicated significance among treatment groups (p < 0.05) (Fig. 1). Pairwise comparisons revealed numerical, non-significant reductions of 0.24, 0.21, 0.25 and 0.35 log₁₀ cfu/g for the postbiotic-PO (A), postbiotic-IC (B), whole culture-PO (C), and whole culture-IC (D) groups, respectively, compared to the control group (G). On the other hand, *Campylobacter* load was significantly reduced by 0.34 log₁₀ in the cells-PO group (E) (p < 0.05), and by 0.78 log₁₀ in the cells-IC group (F), compared to the control (p < 0.001).

### Postbiotics, probiotics and their combinations influence cecal microbial richness

**Fig. 2:**
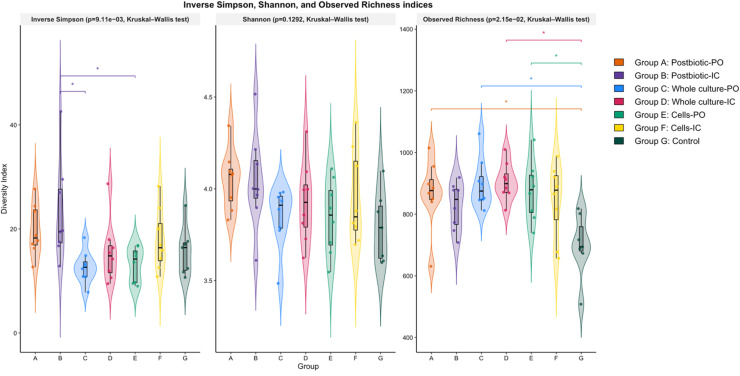
Alpha diversity of the cecal microbiota across treatment groups at week 5. Violin plots illustrate Inverse Simpson, Shannon, and Observed Richness indices. Treatment groups included postbiotic administered orally (Group A, postbiotic-PO) or intracloacally (Group B, postbiotic-IC), whole culture probiotic administered orally (Group C, whole culture-PO) or intracloacally (Group D, whole culture-IC), probiotic cells administered orally (Group E, cell-PO) or intracloacally (Group F, cell-IC), and a non-treated control group (Group G). Boxes represent the interquartile range with median values indicated, while violins depict kernel density distributions of individual samples. Overall group differences were determined using the Kruskal–Wallis test. Post-hoc pairwise comparisons were conducted using Dunn’s test, with significant differences between groups indicated by asterisks (p < 0.05).

The Kruskal–Wallis test revealed significant group effects for both the inverse Simpson (p = 9.11 × 10⁻³) and observed richness (p = 2.15 × 10⁻²) indices, whereas Shannon diversity did not differ (p = 0.129). Post hoc tests revealed that birds receiving postbiotic-IC (B) exhibited significantly higher evenness compared with the whole culture-PO (C) and cell-IC (F) groups (p < 0.05). For observed richness, birds in the postbiotic-PO (A), whole culture groups (C and D), and cells-PO (E) had significantly higher richness than the control (p < 0.05), whereas richness in the postbiotic-IC (B) and cells-IC (F) groups did not differ significantly from the control (G) (Fig. 2).

### Probiotics, postbiotics and their combinations distinctly altered cecal community structure

**Fig. 3:**
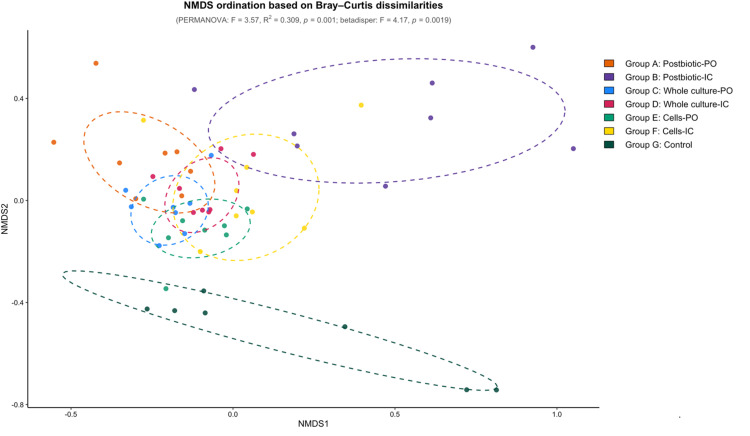
Non-metric multidimensional scaling (NMDS) ordination of cecal microbiota based on Bray–Curtis dissimilarities. Treatment groups included postbiotic administered orally (Group A, postbiotic-PO) or intracloacally (Group B, postbiotic-IC), whole culture probiotic administered orally (Group C, whole culture-PO) or intracloacally (Group D, whole culture-IC), probiotic cells administered orally (Group E, cell-PO) or intracloacally (Group F, cell-IC), and a non-treated control group (Group G). Overall differences in community composition among groups were assessed by PERMANOVA. Heterogeneity of dispersion was detected by betadisper analysis.

Non-metric multidimensional scaling (NMDS) ordination based on Bray–Curtis dissimilarities revealed distinct clustering patterns among treatment groups (PERMANOVA: F = 3.57, R² = 0.309, *p* = 0.001). Pairwise PERMANOVA analysis revealed that the postbiotic-IC group (B) exhibited differences in cecal microbiota structure when compared with the other treatments. It differed significantly from the whole culture groups, C (p = 0.00096) and D (p = 0.00136), the cells-PO group, E (p = 0.00220), and the postbiotic-PO group, A (p = 0.0243). The control group was significantly different from the whole culture groups; C (p = 0.00398) and D (p = 0.00603), as well as the cells-PO (p = 0.0102). In contrast, the postbiotic groups (A and B) and the cells-IC group (F) did not differ significantly from the control (G) (Fig. 3).

### Microbial composition across the treatment groups

The phylum Firmicutes dominated the cecal microbiota in all groups, accounting for 97.46–98.91 % of total relative abundance, with the highest proportion observed in whole culture-PO (C, 98.91 %). The abundance of the phylum Proteobacteria was lower in all treatment groups (0.33-1.45 %) compared to the control group (G, 2.10 %), with the lowest abundance observed in the whole culture-PO group (C, 0.33 %).

**Fig. 4:**
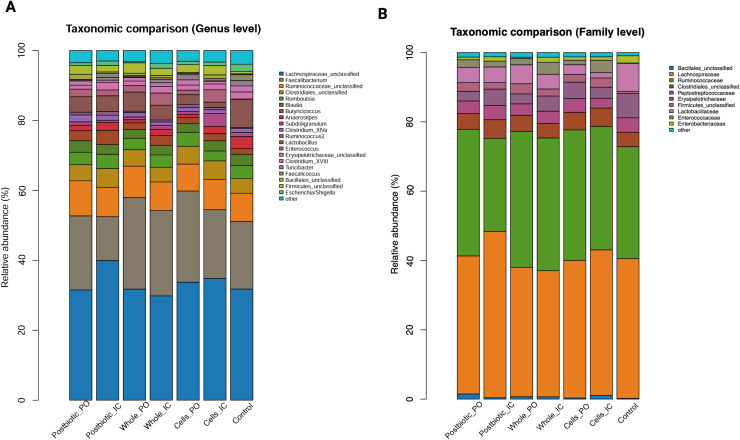
Taxonomic composition of the cecal microbiota across treatment groups at week 5. (A) Genus-level comparison showing the relative abundance of the top 20 most abundant genera across treatment groups, and (B) Family-level comparison showing the relative abundance of the top 10 most abundant bacterial families. Treatment groups included postbiotic administered orally (Group A, postbiotic-PO) or intracloacally (Group B, postbiotic-IC), whole culture probiotic administered orally (Group C, whole culture-PO) or intracloacally (Group D, whole culture-IC), probiotic cells administered orally (Group E, cell-PO) or intracloacally (Group F, cell-IC), and a non-treated control group (Group G). Relative abundances are expressed as percentages of total classified sequences.

Firmicutes-associated genera, especially *Lachnospiraceae_unclassified* and *Faecalibacterium* accounted for the largest fractions of the cecal microbiota (Fig. 4A). *Lachnospiraceae_unclassified* comprised approximately 31.6-39.9 % of sequences across groups, with the highest mean relative abundance in postbiotic-IC (B, 39.9 %) and the lowest in the whole culture-IC group (D, 29.8 %). *Faecalibacterium* had elevated levels in the whole culture groups (C, 26.2 %; D, 24.4 %), and cells-PO (E, 26.1 %) compared to the control (G, 19.3 %). *Ruminococcaceae_unclassified* represented 7.7-10.0 %, with the highest values in the postbiotic-PO group (A, 10.0 %) and lower levels in the cells-PO (E, 7.7 %) and control (G, 8.1 %) groups. The genus *Lactobacillus* ranged from 1.5 % in the cells-IC group (F) to 8.1 % in the control group (G).

*Escherichia-Shigella* abundance was highest in the control group (G, 2.1 %), whereas all treated groups remained below 1.5 %, with the lowest value in the whole culture-PO group (C, 0.3 %). *Faecalicoccus* followed a similar pattern, with the greatest abundance in the control group (G, 1.7 %), intermediate levels in the postbiotic-IC (B, 1.1 %) and cells-IC (F, 0.6 %) groups, and the lowest value in the postbiotic-PO group (A, 0.2 %).

The “other” category, representing genera below the 2 % inclusion threshold, contributed approximately 3.0–3.9 % across groups.

### Differential abundance analysis reveals reduced opportunistic taxa and enrichment of beneficial SCFA-producing genera across probiotic and postbiotic treatments

#### Reduction of opportunistic genera

**Fig. 5:**
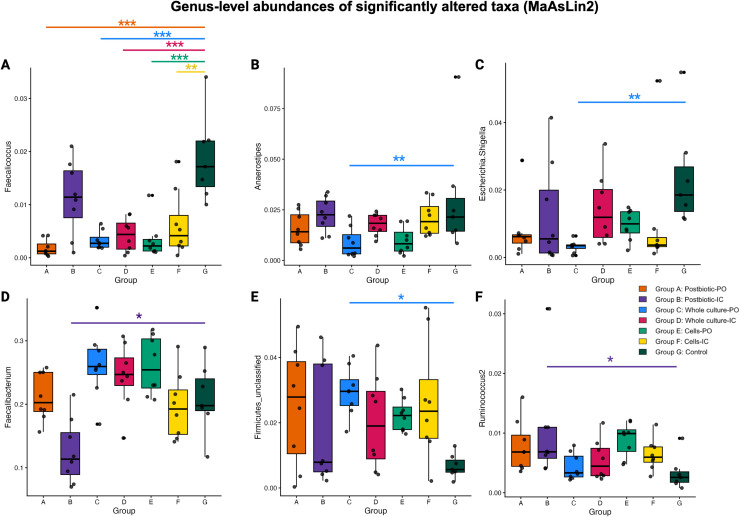
Genus-level relative abundances of taxa identified as significantly associated with treatment by MaAsLin2 analysis. Differential abundance testing was performed using MaAsLin2 with false discovery rate correction (FDR < 0.05). Boxplots display relative abundances across treatment groups: Group A, postbiotic administered orally (postbiotic-PO); Group B, postbiotic administered intracloacally (postbiotic-IC); Group C, whole culture administered orally (whole culture-PO); Group D, whole culture administered intracloacally (whole culture-IC); Group E, probiotic cells administered orally (cells-PO); Group F, probiotic cells administered intracloacally (cells-IC); and Group G, non-treated control. Panels show the following genera: (A) *Faecalicoccus*, (B) *Anaerostipes*, (C) *Escherichia–Shigella*, (D) *Faecalibacterium*, (E) unclassified Firmicutes, and (F) *Ruminococcus*. Boxes represent interquartile ranges with median values indicated, and points represent individual samples. Statistical significance was assessed using MAasLin2 with Benjamini-Hochberg FDR, where asterisks denote FDR-adjusted p values * (*q* < 0.05, ** *q* < 0.01, and *** *q*< 0.001).

Differential-abundance analysis (MaAsLin2) revealed several significant genus-level changes among the treated groups compared to the control group. Opportunistic taxa such as *Faecalicoccus*, was significantly reduced in the postbiotic-PO (coefficient = –0.096, FDR = 5.364e–06), whole culture-PO/IC; C (–0.079, FDR = 9.633e–05) and D (–0.075, FDR = 2.087e–04), and probiotic cells; E (–0.082, FDR = 7.759e–05) and F (–0.064, FDR = 1.918e–03) groups, compared to the control group (G) (Fig. 5A).

Similarly, the whole culture-PO (C) group had significantly lower enrichment with *Escherichia-Shigella* (coefficient = –0.094, FDR = 5.968e–03) compared to the control group (G) (Fig. 5C).

#### Enrichment of beneficial taxa

MaAsLin2 analysis showed distinct changes in the postbiotic-IC group (B), where *Ruminococcus*, a commensal bacterium, was significantly enriched (coefficient = 4.10e–02, FDR = 1.44e–02) (Fig. 5F) and *Faecalibacterium* was significantly reduced (coefficient = –0.115, FDR = 1.225e–02), compared to the control (G) (Fig. 5D).

**Fig. 6:**
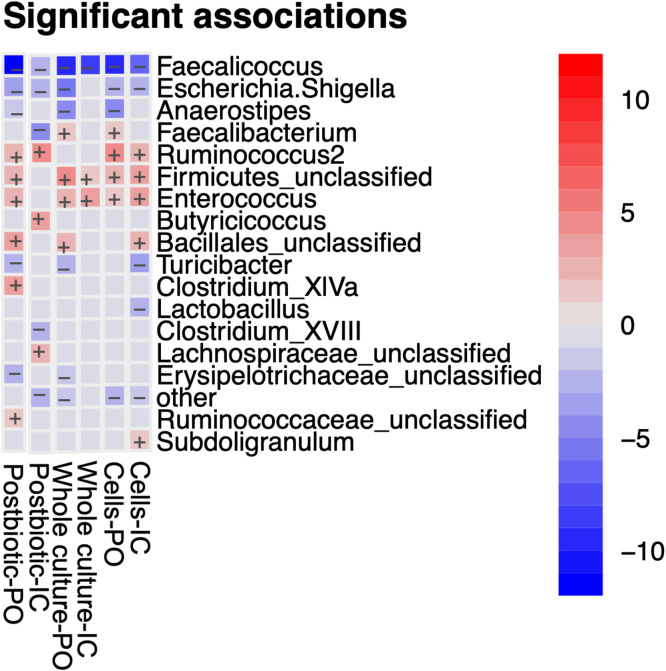
Heatmap of significant genus-level associations identified by MaAsLin2 analysis. Each cell represents the direction and magnitude of association between a bacterial genus and a treatment group relative to the non-treated control (Group G). Color intensity corresponds to the signed −log(*q* value) multiplied by the model coefficient. Red indicates positive associations and blue indicates negative associations relative to the control. Only genera with statistically significant associations after false discovery rate correction (FDR < 0.05) are shown. Treatment groups include postbiotic administered orally (postbiotic-PO), postbiotic administered intracloacally (postbiotic-IC), whole culture administered orally (whole culture-PO), whole culture administered intracloacally (whole culture-IC), probiotic cells administered orally (cells-PO), and probiotic cells administered intracloacally (cells-IC).

In the whole culture-PO group (C), the butyrate-producing genus *Anaerostipes* was significantly reduced (coefficient = –7.51e–02, FDR = 9.74e–03) (Fig. 5B), whereas an unclassified Firmicutes genus was significantly enriched (coefficient = 9.19e–02, FDR = 1.54e–02), compared to the control group (G) (Fig. 5E). Consistent with genus-level abundance, MAasLin2 heatmap visualization revealed distinct treatment-specific microbial signatures (Fig. 6).

## Discussion

Studies on probiotic supplementation for controlling *Campylobacter* in chickens have reported variable outcomes (Helmy et al., 2023; Sharma et al., 2025). For instance, oral supplementation of *L. crispatus* JCM5810 was shown to reduce *C. jejuni* colonization in broiler chickens by more than 2 log₁₀ cfu/g of cecal content (Neal-McKinney et al., 2012). Another study reported that oral administration of *L. salivarius* SMXD51 lowered cecal *C. jejuni* loads by 0.8 log₁₀ cfu/g at day 14 and 2.8 log₁₀ cfu/g at day 35, while also altering the intestinal microbiota composition and enriching beneficial genera such as *Ruminococcus* and *Bifidobacterium* (Saint-Cyr et al., 2017)*.* Conversely, supplementation of different probiotic strains and competitive-exclusion consortia to *C. jejuni*-challenged broilers (Ty et al., 2022) did not influence *Campylobacter* loads but resulted in a distinct shift in the gut microbiota composition, especially among members of the phylum Firmicutes in the treated birds, suggesting that microbiome modification rather than direct pathogen suppression may play a key role in improving gut health. While researchers have attributed the inconsistent findings in probiotic studies to factors such as chicken breed, probiotic strains, and frequency of administration, only a few recent studies have considered the impact of application methods (Sharma, et al., 2025). Moreover, although extensive research has focused on live probiotic cells, little is known about the efficacy of postbiotics.

Hence, the present study was undertaken to evaluate the ability of *Lactobacillus*-based probiotics, postbiotics and their combined whole-culture formulation to reduce cecal *Campylobacter* colonization and modulate the cecal microbiota in broiler chickens when delivered orally or intracloacally. In general, distinct microbial signatures were observed across the treatment groups, with probiotic cell preparations, administered orally or intracloacally, exhibiting enhanced microbial richness and alterations in community composition, in addition to a significant reduction in cecal *Campylobacter* counts. While all other preparations, including postbiotics and the combination of probiotics and postbiotics, induced a variable enhancement of microbial richness and alterations in community structure and composition, a minimal, non-significant reduction in *Campylobacter* counts was observed in these groups.

An interesting finding from this study was that treatment with probiotic cells resulted in significant reductions in cecal *C. jejuni* counts compared to the control, with the intracloacal group achieving the greatest reduction (0.8 log₁₀ cfu/g), followed by the oral group (0.34 log₁₀ cfu/g). Although these reductions are modest, they remain meaningful from a food safety perspective (Koutsoumanis et al., 2020). Recent quantitative microbial risk assessment reports suggest that a 2 log₁₀ reduction in broiler cecal *Campylobacter* concentrations would lower the relative risk of human campylobacteriosis from broiler meat by around 40% (Koutsoumanis et al., 2020; Nauta et al., 2022). Moreover, the slightly better performance of the intracloacal route over oral gavage aligns with previous reports showing enhanced efficacy of probiotics when delivered directly to the lower gut (Arsi et al., 2015a). This may be explained by bypassing the acidic and enzymatic barriers of the upper gastrointestinal tract, allowing a larger number of viable cells to reach the ceca, the main site of *Campylobacter* colonization (Arsi et al., 2015a). While intracloacal delivery is not practical at a commercial scale, its superior performance highlights the importance of targeted delivery. In view of this, continuous dosing and/or protective encapsulation techniques may be necessary to ensure that a large number of probiotic cells reach the lower gut when administered orally.

Although *Lactobacillus* treatment produced only a modest reduction in *C. jejuni* colonization, supplementation of these beneficial bacteria may still confer other benefits, such as modulating the gut microbiome and enhancing growth performance. This study primarily focused on the impact of *Lactobacillus* cells and their byproducts on microbiome diversity and composition in chickens challenged with *Campylobacter*.

Phylum-level taxonomic analysis revealed a reduced relative abundance of Proteobacteria in all treatment groups compared to the control group, suggesting that this treatment mitigated potential microbial shift and proliferation of opportunistic Gram-negative taxa, such as *Campylobacter, E. coli*, and *Salmonella*. Since *Campylobacter* belongs to the Proteobacteria lineage, these observations further confirm the reduction in *Campylobacter* counts in the treatment groups compared to the control group.

Family-level taxonomic analysis revealed a similar community composition, dominated by families *Lachnospiraceae* and *Ruminococcaceae*, in the groups treated with probiotic cells either orally or intracloacally. Community structure, as visualized by NMDS ordination, revealed that these two groups clustered closely together, indicating similar microbial community compositions that were distinct from the control group. Furthermore, the heatmap of significant associations aligned with this pattern, with both groups showing positive associations with butyrate-producing genera such as *Ruminococcus* and *unclassified Firmicutes*, and negative associations with *Faecalicoccus* and *Escherichia/Shigella*, suggesting that *Lactobacillus* cells can exert selective pressure against opportunistic taxa in the gut.

The group receiving the combined probiotics and postbiotics orally also demonstrated favorable modulation of the microbiome. Differential abundance analysis showed a significant reduction of *Faecalicoccus* in this treatment group. Similarly, *Faecalicoccus* was also significantly lowered in groups receiving postbiotics or probiotics orally. Studies have noted that *Faecalicoccus* tends to increase under dysbiotic or inflammatory conditions (Forbes et al., 2018). Additionally, this treatment group was the only group with a statistically significant decrease in the genus *Escherichia-Shigella* (grouping reads that could belong to either *E. coli* or *Shigella* due to 16S similarity), with a relative abundance of around 0.3% compared to the control (2.1%). This likely reflects the direct antagonistic effects of the probiotics and their byproducts, aligning with previous studies reporting a suppression of *Escherichia-Shigella* growth through competitive exclusion and secretion of antimicrobial compounds (Arreguin-Nava et al., 2019; Jha et al., 2020; Khalid et al., 2025; Ma et al., 2018; van der Klein et al., 2024). Additionally, an enrichment of unclassified Firmicutes genera was observed in the combined treatment group, underscoring the unique benefit of pairing live probiotic cells with their postbiotic byproducts. These unclassified Firmicutes likely represent uncultured or under-characterized taxa with functional roles in gut homeostasis, potentially reflecting enhanced substrate availability or niche compatibility (Dias et al., 2025; Guo et al., 2024).

On the other hand, administration of postbiotics intracloacally significantly enriched both *Faecalibacterium* and *Ruminococcus* in treated birds compared to the control. Faecalibacterium is one of the dominant genera in the chicken cecum and plays a role in producing SCFAs, maintaining gut barrier integrity, and regulating inflammation (Eeckhaut et al., 2011; Oakley et al., 2014; Polansky et al., 2016; Yan et al., 2017)*.* Similarly, *Ruminococcus* is another SCFA-producing bacterium known for breaking down complex polysaccharides and generating beneficial byproducts like acetate, propionate, and butyrate (La Reau et al., 2016). The high abundance of this genus in the postbiotic-treated birds suggests that these byproducts may have promoted the growth of fiber-degrading microbes well adapted to the gut environment. As a strictly anaerobic genus, the enrichment of *Ruminococcus* is best suited to this delivery method. A higher presence of such butyrate-producing bacteria can support gut health by lowering cecal pH, inhibiting pathogenic taxa, while nourishing enterocytes and strengthening the gut barrier function (Ríos-Covián et al., 2016).

Additionally, the same postbiotic group exhibited a significantly higher microbial evenness, as indicated by the inverse Simpson index. These findings suggest that delivering *Lactobacillus* byproducts directly to the hindgut can normalize the community structure, perhaps by suppressing overabundant taxa and encouraging a more uniform distribution. While each intervention created a unique shift in the microbiota composition, the intracloacal postbiotic group formed a separate cluster in the NMDS plot that differed significantly from the other groups, suggesting its more pronounced impact on cecal community structure. Supporting these observations, earlier studies reported that *Lactobacillus* postbiotics can exert selective pressures on the microbiota by lowering gut pH or providing readily fermentable substrates, thereby altering the growth of various taxa (Aguilar-Toalá et al., 2018; Mosca et al., 2022; Scott et al., 2022; Thorakkattu et al., 2022).

*Campylobacter* sequences were not detected in the 16S rRNA amplicon dataset, likely because their abundance fell below the detection threshold. Amplicon-based sequencing often fails to capture low-abundance taxa, particularly when their relative abundance is below 0.1 % of the total community (Pollock et al., 2018; Quince et al., 2017). However, culture-based enumeration confirmed the presence of *Campylobacter* in cecal contents, indicating that the bacterium persisted at levels below the detection limit of 16S rRNA sequencing. Similar findings have been noted in previous poultry microbiome studies (Awad et al., 2016; Hermans et al., 2011). Nonetheless, the incorporation of mock microbial communities and spike-in controls with *Campylobacter* is warranted, as it ensures more reliable quantification and minimizes potential biases in 16S rRNA sequencing analyses.

While microbial-based interventions have demonstrated the capacity to shift gut microbiota, achieving consistent *Campylobacter* suppression *in vivo* remains a challenge (Taha-Abdelaziz et al., 2018b; Ty et al., 2022). For instance, in a broiler challenge model, supplementation with two microbial consortia yielded lower *C. jejuni* burdens compared to the bacitracin-treated group but were not significantly different from the untreated controls (Ty et al., 2022). Despite no appreciable decrease in *C. jejuni* levels, each intervention induced a distinct cecal microbial signature (e.g., *Bacteroidaceae* and *Rikenellaceae*) enriched under different treatments, suggesting that microbiome modulation rather than direct pathogen killing may contribute to a healthier gut.

Overall, the method of delivery (oral vs. intracloacal) together with the form of probiotic product (whole culture, cells, or cell-free supernatant) appears to significantly influence outcomes. Such information can guide practical applications; for example, although intracloacal administration of *Lactobacillus* cells outperformed oral delivery, it may be less practical on a commercial scale compared to incorporating probiotics in feed or water. Further studies are required to investigate whether continuous *Lactobacillus* administration via drinking water, feed, or microencapsulation would lead to greater reduction in *C. jejuni* counts.

Postbiotics, on the other hand, offer an interesting avenue as they contain no live microbes and thus avoid issues of viability. In our study, although not all probiotic and postbiotic formulations achieved statistically significant reductions in cecal *Campylobacter* loads, all treatments contributed to appreciable modulation of the gut microbiota.

A key limitation of this study is the lack of functional metagenomic or metabolomic profiling, which limits the ability to directly link microbial taxonomic changes with metabolite production and pathogen suppression. Future research should integrate optimized probiotic formulations with functional omics approaches and mechanistic analyses to better understand the interactions driving microbiome modulation and *Campylobacter* control.

In addition, the administered *Lactobacillus* probiotic strains were not directly recovered from cecal contents using culture-based or strain-specific molecular methods. As a result, probiotic survival, persistence, and colonization following oral or cloacal administration were not assessed. Since 16S rRNA gene amplicon sequencing does not provide sufficient taxonomic resolution to distinguish administered probiotic strains from closely related endogenous taxa, comparisons between delivery routes and probiotic formulations in this study are based on ecological outcomes rather than strain-level persistence.

In conclusion, this study demonstrates that supplementation with poultry-derived *Lactobacillus* cells, their postbiotics, or a combination of both beneficially modulates the gut microbiota by increasing the abundance of members of the phylum Firmicutes and reducing the abundance of Proteobacteria, which includes *Campylobacter*. Importantly, *Lactobacillus* cells provided an added advantage by significantly decreasing *Campylobacter* colonization. These findings suggest the potential use of *Lactobacillus-*based probiotics strategies to reduce *Campylobacter* burden in broiler production and promote a more favorable cecal microbiome.

## Data availability statement

The 16S rRNA sequencing data and metadata generated in this study have been deposited in the NCBI Sequence Read Archive (SRA) under BioProject accession number PRJNA1322503.

## Funding declaration

This research was supported by the South Carolina Department of Agriculture (SCDA) under grant number 2017055 and by the Clemson University Research Foundation (CURF) under grant number 2017099. Additional support was provided by the USDA 10.13039/100005825National Institute of Food and Agriculture Hatch Project SC-1700628 (Accession Number 7004405, Technical Contribution No. 7505). Publication support was also provided by the Clemson University Libraries Open Access Publishing Fund. Additionally, this research was made possible, in part, with support from the Clemson University Genomics and Bioinformatics Facility, which receives support from the College of Science and two Institutional Development Awards (IDeA) from the National Institute of General Medical Sciences of the National Institutes of Health under grant numbers P20GM146584 and P20GM139769.

## CRediT authorship contribution statement

**Shreeya Sharma:** Writing – review & editing, Writing – original draft, Software, Methodology, Investigation, Formal analysis, Data curation. **Hosni Hassan:** Writing – review & editing, Writing – original draft, Visualization, Validation, Resources, Conceptualization. **Khaled Abdelaziz:** Writing – review & editing, Writing – original draft, Visualization, Validation, Supervision, Resources, Project administration, Investigation, Funding acquisition, Data curation, Conceptualization.

## Disclosures

The authors declare no competing interests.
